# Fuzzy Comprehensive Evaluation Assistant 3D-QSAR of Environmentally Friendly FQs to Reduce ADRs

**DOI:** 10.3390/ijerph16173161

**Published:** 2019-08-29

**Authors:** Zhixing Ren, Yingwei Wang, Haihong Xu, Yufei Li, Song Han

**Affiliations:** 1 College of Forestry, Northeast Forestry University, No. 26 Hexing Road, Harbin 150040, China; 2 Appraisal Center for Environmental & Engineering Ministry of Ecology and Environment, No. 28 Beiyuan Road, Beijing 100012, China

**Keywords:** ADRs, fuzzy comprehensive evaluation, 3D-QSAR method

## Abstract

Most studies on adverse drug reactions (ADRs) of fluoroquinolones (FQs) have focused on the mechanisms of single ADRs, and no quantitative structure–activity relationship (QSAR) method studies have been carried out that combine several ADRs of FQs. In this study, an improved three-dimensional (3D) QSAR method was established using fuzzy comprehensive evaluation. This method could simultaneously consider three common ADRs of FQs using molecular parameters. The improved method could comprehensively predict three ADRs of FQs and provide direction for the development of new drugs with lower ADRs than the originals. According to the improved method, 48 derivatives with lower ADRs (decreased by 4.86% to 50.92%) were designed from pazufloxacin. Three derivatives with a higher genotoxicity, higher photodegradation, and lower bioconcentration than pazufloxacin were selected using the constructed QSAR methods of the FQs. Finally, three traditional 3D-QSAR methods of single ADR were constructed to validate the improved method. The improved method was reasonable, with a relative error range of 0.96% to 4.30%. This study provides valuable reference data and will be useful for the development of strategies to produce new drugs with few ADRs. In the absence of complementary biological studies of these adverse drug reactions, the results reported here may be quite divergent from those found in humans or experimental animals in vivo. One major reason for this is that many adverse drug reactions are dependent upon enzyme-catalyzed metabolic activation (toxication) or on non-enzymatic conversion to toxic products and are not due to the parent drug moiety.

## 1. Introduction

**Figure 1:**
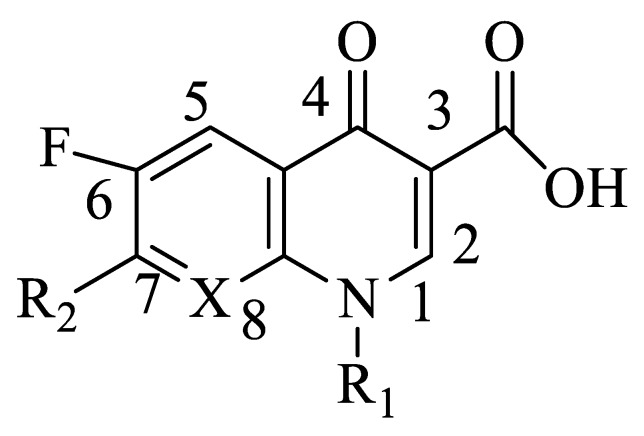
Maternal structure of fluoroquinolones (FQs). R_1_ is a hydrocarbon group usually, R_2_ is usually a piperazine ring, X is carbon or nitrogen.

**Figure 2:**
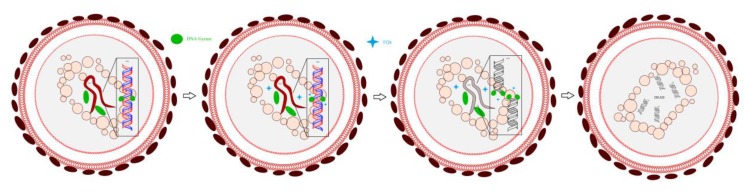
Mechanism of fluoroquinolone action in bacterial cells.

Quinolone antibiotics are a class of antibacterial agents with a wide range of activity. Fluoroquinolones (FQs, Figure 1) have broad-spectrum and strong antibacterial activity and good tissue distribution [1]. Presently, there are more than a dozen common FQs in clinical use. These drugs target bacterial DNA gyrase and inhibit bacterial DNA helix-promoting enzymes so that DNA cannot control the transcription and translation of RNA and proteins. This results in degradation of chromosomal DNA by nucleic acid exonuclease, which affects the normal shape and function of DNA and causes irreversible damage to chromosomes. Finally, DNA replication is blocked, and bacterial cells no longer divide, which is the bactericidal mechanism of FQs [2] (Figure 2).

FQs have broad-spectrum antimicrobial activity, widespread distribution in vivo, and high tissue concentrations. They are widely used in human health, aquaculture, and animal husbandry. Consequently, they continuously enter the environment, where they are pseudo-persistent pollutants [3]. FQs are relatively safe antibiotics that are undergoing rapid development. However, with widespread clinical application, reports of adverse drug reactions (ADRs) are increasing. These reactions can involve nerves, blood, urine, the respiratory system, liver, kidneys, muscles, and bones [3,4,5]. There are three common ADRs to FQs: Convulsive toxicity, gastrointestinal toxicity, and cardiotoxicity.

Part of the FQ structure is similar to that of antagonists of the central nervous system inhibitor, γ-aminobutyric acid (GABA), thus it can interfere with GABA binding to receptors, inhibit GABA activity, produce central excitatory effects, release excitatory thresholds, and even induce convulsions. The incidence of these effects’ ranges from 1% to 17% [6]. Experiments have confirmed that FQs can contact GABA receptors and hinder their binding to GABA [7], which triggers central nervous system responses, such as nausea, vomiting, tremors, agitation, convulsions, and palpitations. Adverse reactions in the gastrointestinal system, such as nausea, vomiting, abdominal pain, and diarrhea, are one of the most common reactions caused by FQs, and the incidence rate ranges from 2% to 16% [8]. FQs can stimulate the secretion of gastric acid and pepsin, inhibit the secretion of mucus and bicarbonate, inhibit the synthesis of cyclooxygenase (COX), and decrease prostaglandin, which has a protective effect on the gastric mucosa. They can also weaken the defense mechanism of gastric acid and directly damage gastrointestinal mucosa, which causes abdominal pain, nausea, and diarrhea [9,10,11]. The adverse cardiac effects of FQs are caused by the fact that certain FQs can bind to potassium channel proteins on the myocardial cell membrane to prolong the QT interval in electrocardiograms [12] and also prolong the QTc. This can produce malignant ventricular arrhythmias, such as polymorphic ventricular tachycardia, torsade de pointes, and ventricular fibrillation [13].

**Figure 3:**
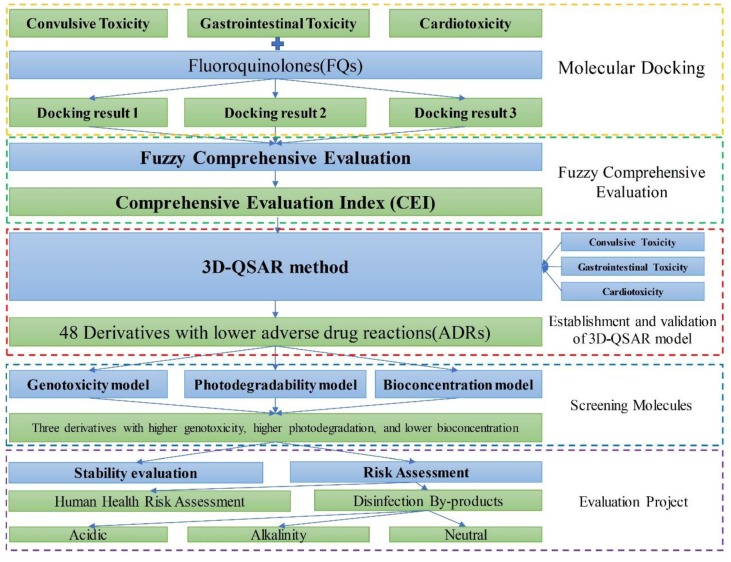
Workflow chart.

In this paper, representative FQs were selected for analysis. For each FQ, the quantitative relationship between the molecular structure and comprehensive evaluation index (CEI) was studied using a fuzzy comprehensive evaluation method, and a stable and predictive model was established. The influences of molecular fields on the FQs in the three-dimensional (3D) equipotential diagram of the model were used to design some derivatives with reduced CEI, taking pazufloxacin (PAZ) as a template. Simultaneously, to better understand the environmental behavior of FQs and design PAZ derivatives, the genotoxicity, bioconcentration, and photodegradability were evaluated. Then, three single-factor 3D-quantitative structure–activity relationship (QSAR) models of FQs were constructed to validate the multi-factor 3D-QSAR model of FQs. Chlorine disinfection by-products of PAZ and its derivatives were also simulated, and their risks predicted using the model. The model can be used to predict the CEI of FQ homologs and their derivatives, design FQs with lower ADRs (decreased by 4.86% to 50.92%), and evaluate the environmental behavior of FQs. This research provides important theoretical support for studies of FQs and their chlorine disinfection by-products. The workflow chart can be seen in Figure 3.

## 2. Materials and Methods

### 2.1. Characterization of the ADRs of FQs

To characterize the ADRs of FQs, Discovery Studio 4.0 software (BIOVIA, San Diego, CA, USA) was used with the molecular docking method. The protein structure was divided into donor and receptor parts. The receptor molecule was a biological protein representing ADRs towards the GABA receptor, COX, and hERG potassium channel protein. The structures of the three binding protein were obtained from the Protein Data Bank and had codes of 3IP9, 3N8V, and 2L0W, respectively. The donor molecules were 29 FQs. The Lib Dock module in Discovery Studio 4.0 was used for molecular docking calculations, Find Sites from the Receptor Cavities tool under the Define module was used to find possible binding sites in the receptor, and binding sites were modified and defined. Finally, the ligand molecules were incorporated into the binding cavity of the formed protein to interface with the ligand protein, and the change in binding ability was analyzed using scoring values [14,15].

### 2.2. Construction of a 3D-QSAR Model Using the Fuzzy CEI of ADRs of FQs

#### 2.2.1. Construction of a Fuzzy Comprehensive Evaluation System for ADRs of FQs

Comprehensive evaluation refers to the overall and holistic evaluation of the target system described by multi-attribute architecture. That is, for the entire evaluation target, an evaluation value (e.g., a CEI) is given to each evaluation object by a selected method according to certain conditions [16].

Through analysis of the docking scores of the FQs with the GABA receptor, COX, and hERG potassium channel protein, three ADR toxicity indicators (i.e., convulsive toxicity, cardiotoxicity, and gastrointestinal toxicity) were determined for the organism consuming FQs. The results of the three ADRs were used for an overall evaluation of the ADRs of the drugs on the organisms, and the CEI was obtained. In the comprehensive evaluation process, the incidence of an ADR is affected by various ambiguous factors. Therefore, the method of combining fuzzy theory with the classical comprehensive evaluation method for comprehensive evaluation is called fuzzy comprehensive evaluation. There are two main steps in the mathematical model of fuzzy comprehensive evaluation.

The first of these is single-factor evaluation. In this study, a large-scale membership function was selected for single-factor evaluation to obtain an evaluation matrix:

(1) $$R=(r_{ij})\;=\;\left[\begin{array}{llll} r_{11} & r_{12} & \cdots & r_{1j}\\ r_{21} & r_{22} & \cdots & r_{2j}\\ \vdots & \vdots & & \vdots \\ r_{i1} & r_{i2} & \cdots & r_{ij} \end{array}\right],$$

where $r_{ij}$ is the priority of the *j*-th molecule with respect to the *i*-th ADR [17], and is given by the following Equation (2):

(2) $$r_{ij}=\frac{c_{ij}-\min\left\{c_{ji}\right\}}{\underset{j}{max}\left\{c_{ji}\right\}-\underset{j}{min}\left\{c_{ji}\right\}}.$$

The second step is fuzzy comprehensive evaluation. In this step, the weighting coefficient is given by the incidence rate of each ADR as follows: $A=\left(a_{1},a_{2},a_{3}\right)$. Using the weighted average model for comprehensive evaluation, the evaluation results for the three ADR indicators are given by C=A R [18].

#### 2.2.2. Structure Optimization and Superposition of FQ Molecules

**Figure 4:**
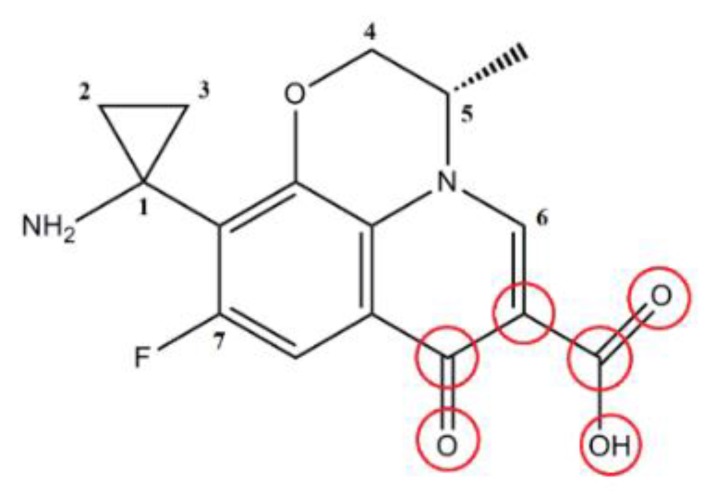
Selecting the target compound to align with the common framework.

**Figure 5:**
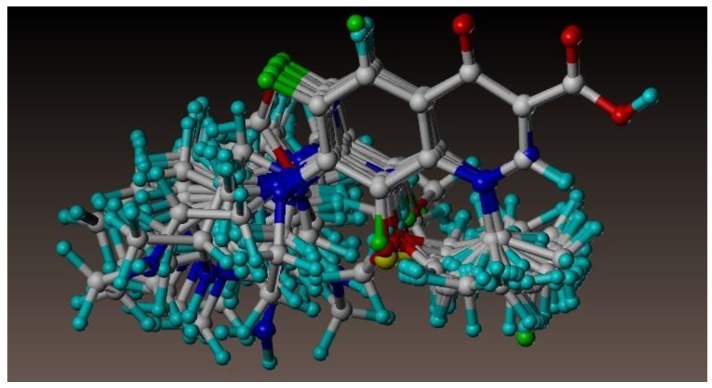
The result of all the molecules overlapping.

The molecular structure drawn directly in SYBYL-X 2.0 software (Tripos, Princeton, NJ, USA) is not the most stable conformation of the molecule. In the case of unknown receptors, the conformation of the molecule at the lowest energy is generally selected. Energy optimization of the molecule generally uses the Tripos force field in SYBYL-X software. The field and molecular program Minimize was used, and Gasteiger–Huckel charges were loaded on the molecule. The Powell energy gradient method was used for molecule optimization, with the maximum number of optimizations set as 10,000, the energy convergence limited to 0.005 kJ/mol, and the rest of the parameters set to default values. The results were stored in a database for stacking. In this paper, PAZ, which had the largest CEI, was selected as the target molecule, and the pharmacophore characteristic elements in the labeled area shown in Figure 4 were used as the basic framework for overlapping. The Alignment Database module was used for automatic overlapping to ensure that the orientation of each molecular field was consistent. The results showed that all molecules overlapped well (Figure 5).

#### 2.2.3. Construction of a 3D-QSAR Model Using Multiple ADRs of FQs

In this paper, 3D-QSAR analysis was performed using SYBYL-X 2.0 software [19]. To ensure structural diversity and a wide distribution of compounds, the calculated data were taken from the CEI values of the 29 FQs. According to a ratio of about 3:1, 22 compounds were randomly selected as a training set and eight compounds were used as a test set. The target molecules were in both the training and test sets [20]. After repeated random tests, the 3D-QSAR model was finally established.

Comparative molecular field analysis (CoMFA) analysis can be completed in the QSAR module of SYBYL-X 2.0. When calculating the parameters of the CoMFA field, the molecular force field is electrostatic (E) and steric (S). The dielectric constant is related to distance. The threshold value is 125.4 kJ/mol. The other parameters were set to the default values of the system. Finally, the parameters of the molecular force field were obtained. The CEI values of 22 FQs in the training set were sequentially entered in the training table, and the parameters of the CoMFA field were automatically calculated using Autofill in SYBYL-X 2.0. The relationship between the structure of the target compound and biological activity was established by partial least squares analysis. When using partial least squares analysis, the training set compounds are cross-validated first using the leave-one-out method to obtain the cross-validation coefficient (*q*^2^) and the optimal principal component number (*n*). Then, through regression analysis of non-cross validation regression (no validation), the non-cross validation coefficient (*r*^2^), standard error of the estimate (*SEE*), and *F*-test value were obtained, and the CoMFA model was established.

### 2.3. Feasibility of Molecular Modification of FQs and Stability Evaluation of the Derivatives

Theoretical calculations using density functional theory with B3LYP/6-31G (d) in Gaussian 09 were used to optimize the structures of FQs and their derivatives in the environment and calculate the transition state (TS) and substitution reaction energy barrier (∆*E*) for the derivatization.

## 3. Results and Discussion

### 3.1. Comprehensive Evaluation of the Three ADR Indicators of FQs

Before docking, protein receptor molecules were obtained from the PDB database, and the PDB ID of GABA receptor, cyclooxygenase, and hERG potassium channel protein was 3IP9, 3N8V and 2L0W. The above ID were chosen because the proteins were more suitable for our research.

The convulsion toxicity, gastrointestinal toxicity, and cardiotoxicity were obtained from the docking results of the 29 FQs and GABA receptor, hERG potassium channel protein, and cyclooxygenase, and the incidences of ADRs with various antibiotics. The weighting coefficient of the ADRs was *A* = (50%, 40%, and 10%). Finally, the three ADRs were evaluated by fuzzy comprehensive evaluation (Table 1).

The fuzzy CEI had a linear relationship with the indicators of the three ADRs, and a high degree of agreement (Table 1). Consequently, further evaluations in this study used the CEI.

### 3.2. Construction and Evaluation of the 3D-QSAR Model for ADRs of FQs Using the Fuzzy CEI

#### 3.2.1. Analysis and Evaluation of the FQs CoMFA Model

Since there is no relevant researcher to carry out QSAR research on ADRs of multiple FQs, the author, based on the CEI index obtained by the above comprehensive evaluation method, established a 3D-QSAR model based on the combination of several ADRs of FQs.

The cross-validation coefficient (*q*^2^) of the CoMFA model based on the fuzzy comprehensive evaluation method was 0.560 (>0.5), and the optimum principal component number (*n*) was 10. These results indicate that the model has good prediction ability. In addition, the non-cross-validation coefficient (*r*^2^) was 0.999 (i.e., >0.9 and close to 1.0), indicating that this model has a good fit. In the model scrambling stability test, the standard error of prediction of the cross-validation of the experimental and predictive values of the CoMFA model was 0.215, and the external predictive set cross-checking coefficient (*r*^2^*_pred_*) was 0.692 (i.e., >0.6). In the scrambling stability text, we calculated the standard error of prediction (SEP), the perturbation prediction (Q^2^_ext_), the cross-validated standard error of prediction (cSDEP) as a function of the correlation coefficient between the true values (y) of the dependent variables and the perturbed values (y’) of the dependent variables, and the slope of Q2 with respect to the correlation of the original dependent variables against the perturbed dependent variables (dq2/dr2yy).These results indicate that the constructed 3D-QSAR model has high external predictive power (Table 2) [21]. In the CoMFA model, the contribution rates of the stereo field and the electrostatic field were 55.5% and 44.5%, respectively, indicating that the spatial and electronic distributions had significant effects on the CEIs of the FQs.

The external Q^2^_ext_ for the test set was determined with the following equation [22]:

(3) $$Q_{ext}^{2}=1-\frac{\sum \left(y_{i}-{\tilde{y}}_{i}\right)}{\sum \left(y_{i}-\bar{y}\right)},$$

where $y_{i}$ and ${\tilde{y}}_{i}$ are the observed and calculated response values, respectively; and $\bar{y\;}$ is the averaged value for the response variable of the training set.

CoMFA methods are widely used 3D-QSAR techniques that are useful to relate any variation of an experimentally determined parameter (dependent variables), related to a set of molecules, with respect to specific descriptors, which are considered as independent variables. In particular, the steric and electrostatic fields were calculated by CoMFA analysis, respectively [23].

#### 3.2.2. Validation of the FQs CoMFA Model

**Figure 6:**
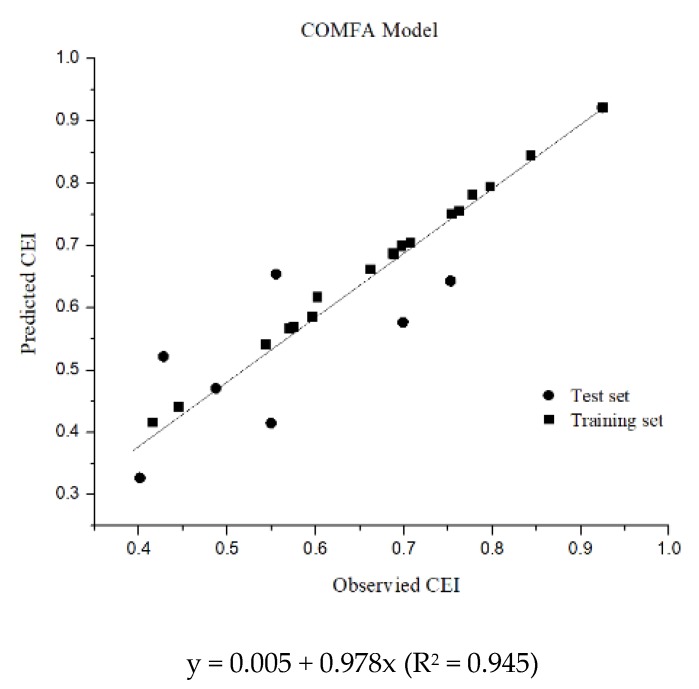
Correlation between experimental values and predicted values of CEI in the CoMFA model.

To test the internal predictive ability of the model, the CEIs of the original 29 FQs were predicted using the CoMFA model using the CEI. The difference between the predicted value of the model and the actual experimental data ranged from −3.31% to 2.78%, and the error was negligible and within ±5%. Activity prediction of the test set molecules in Table 2 was performed using the constructed CoMFA model to verify the accuracy of the constructed model. As shown in Figure 6, the predicted values were linearly correlated with the experimental values and showed good agreement. All the data were concentrated near the trend line with a slope of 0.978. The correlation coefficient of linear fitting between the predicted values and the experimental values reached 0.945, which indicated that the model had high internal prediction ability and could be used to predict the CEIs of FQs. The experimental and predicted values of fluoroquinolones through the CoMFA model can be seen in Table 3.

### 3.3. Determination of Single and Double Substitution Sites and Substituent Groups of FQ Derivatives Using Contour Maps

Taking PAZ as a template, a block diagram of different colors was constructed around the molecule to indicate the influence of stereo, electrostatic fields on the CEI values of the FQs. The area type used for all 3D equipotential maps is the standard deviation coefficient (stdev *-coeff), with a support contribution of 80% in the default value and 20% unsupported contribution. For the stereo field, green indicates that the introduction of a bulky group can increase the activity of the compound, and yellow indicates that the introduction of a bulky group can reduce the activity of the compound. For the electrostatic field, blue shows an increase in the band. A positively charged group increases the activity of the compound, and red indicates that an increase in electronegative groups increases the activity of the compound.

**Figure 7:**
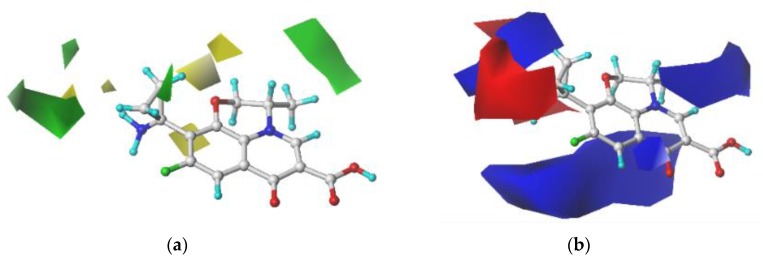
Contour maps of the CoMFA model, steric fields (**a**); electrostatic fields (**b**).

In the CoMFA model contour maps (Figure 7), there is yellow at the end of the substituent at the 1-position. This indicates that the introduction of a bulky group at the 1-position will decrease the CEI of the FQ. For the electrostatic field, there is red at the end of the substituents at the 1- and 2-positions. This indicates that the introduction of a positively charged group at these positions will decrease the CEI of the FQs. There is blue at the end of the substituent at the 5-position, which indicates that the introduction of a negatively charged group at this position will decrease the CEI of the FQ. On the basis of these results, we chose to modify the 1- and 5-positions with the following 12 groups: –CH_3_, –H, –C_2_H_5_, –C_2_H_3_, –C_2_H, –CO; –OH, –COOH, –SH, –F, –Cl, and –Br. This produced 48 FQ derivatives.

### 3.4. Comprehensive Evaluation, Persistent Organic Pollutant Characteristics, and Stability Evaluations of FQ Derivatives with Three Common ADRs

#### 3.4.1. Fuzzy Comprehensive Evaluation of FQ Derivatives and Three Common ADRs

A total of 48 FQ derivatives were produced by single and double substitutions. The comprehensive evaluation results of the three common ADRs of these 48 FQ derivatives were predicted (Table 4).

The predicted results of the 48 PAZ derivatives were lower than the CEI values of PAZ, with reductions ranging from 4.86% to 50.92% (Table 4). The effect of reducing the fuzzy CEI of individual PAZ derivatives is not significant enough, but it can provide a theoretical reference for the replacement of FQs.

#### 3.4.2. Evaluation of the Persistent Organic Pollutant Characteristics of FQ Derivatives

To evaluate their persistent organic pollutant characteristics of the 48 PAZ derivatives with low ADRs, their genotoxicities (pLOEC), bioconcentration (log*K_ow_*), and photodegradability (log*t*_1/2_) of their persistent organic pollutants (POPs) characteristics were predicted using the known QSAR models in Table 5 [24,25,26].

Among the 48 PAZ derivatives, 28 derivatives, including derivative-1, showed an increase of 0.00% to 7.48% compared with the template molecule (Table 5). For further predictions, these 28 derivatives were screened using a photodegradability model. Twenty-four PAZ derivatives with high genotoxicity and photodegradability, such as derivative-1, were obtained. The photodegradability of these derivatives increased by 1.79% to 15.87% compared with PAZ. Through prediction and screening with a bioconcentration model, 1-methyl-PAZ, 1-hydrogen-PAZ, and 1-ethyl-PAZ were finally identified as environmentally friendly derivatives with low ADRs. For these derivatives, the bioconcentration decreased by 74.65% to 87.72% compared with PAZ.

#### 3.4.3. Single-Factor Validation of the ADRs of the FQ Derivatives

In this paper, three common ADRs of FQs were evaluated by fuzzy comprehensive evaluation. In this evaluation system, a weighting of 50%, 40%, or 10% was assigned to each of the three ADRs according to their incidences. However, further research is required to verify whether this subjective weighting is reasonable.

Therefore, three 3D-QSAR models corresponding to the three ADRs were constructed using the initial docking results of the 29 FQs and the corresponding proteins of the three ADRs. The single-factor effects of the three common ADRs were predicted and evaluated for three derivatives. The results were used to verify the rationality of the subjective weighting method. The predicted results are shown in Table 6.

As shown in Table 6, the CEIs of the three derivatives decreased by 29.84% to 37.51%. With a weighting of 50%, the corresponding reduction range for convulsive toxicities was 14.92% to 18.76%. Similarly, the ranges for the reductions in gastrointestinal toxicity (40%) and cardiotoxicity (10%) were 11.95% to 15.01% and 2.98% to 3.75%, respectively. In a comparative evaluation of convulsive toxicity and gastrointestinal toxicity, the relative errors between the comprehensive evaluation parameters and the single-factor evaluation parameters of the three derivatives were less than 5%. However, in an evaluation of cardiotoxicity, the relative error was large (12.42%–32.78%) because the weighting was only 10%, which accounted for only a small proportion and resulted in relatively large errors in the cardiotoxicity comparison.

The single-factor effect verification results of convulsive toxicity and gastrointestinal toxicity, which accounted for 90% of the weighting of the comprehensive evaluation system, were reasonable. This verified that the weighting given according to the ADR incidence was reasonable for establishing the fuzzy comprehensive evaluation system. At the same time, the rationality of the multi-factor 3D-QSAR model of FQs and the fuzzy evaluation method was verified.

#### 3.4.4. Stability Evaluation of FQ Derivatives

To further realize derivatization of the PAZ molecule, we characterized the stabilities of 1-methyl-PAZ, 1-hydrogen-PAZ, and 1-ethyl-PAZ in the environment.

**Figure 8:**
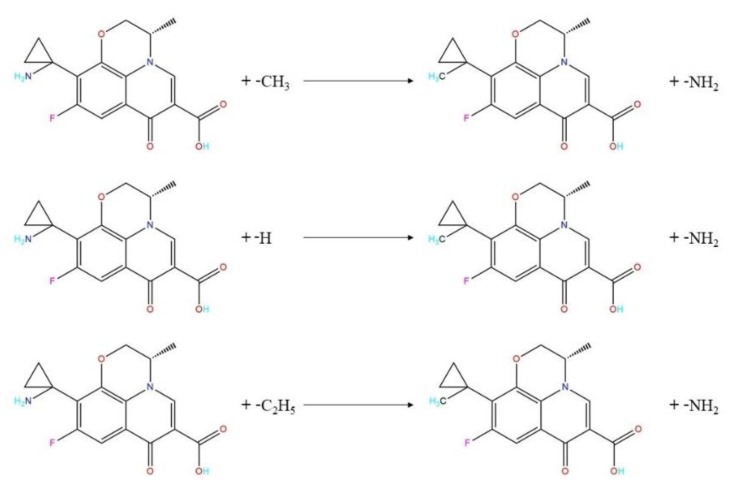
The substitution reaction paths of PAZ.

Taking PAZ as a template, the substitution reaction pathways of PAZ with –CH_3_, –H, and –C_2_H_5_ are shown in Figure 8. The Gibbs free energy change (Δ*G*) for each substitution reaction was calculated using Equation (4) to determine the likelihood the reaction occurring:

(4) ΔG=∑G(Product)−∑G(Reactant).

In addition to evaluating the likelihood of the substitution reactions, it is necessary to compare the difficulties of the three substitution reactions of the PAZ molecule. Density functional theory was used to calculate the TS and reaction energy barrier (Δ*E*) of each substitution reaction. The TS [27] is verified by the inner coordinates, and there must be only one virtual frequency [28]. The reaction energy barrier was calculated using Equation (5). The calculation results are shown in Table 7:

(5) ΔE=E(TS)−∑E(Reactant).

The positive frequencies of the three FQ derivatives were greater than zero. Each positive frequency represents the lowest energy in a certain dimension, which indicates that the three FQs derivatives have stable structures and can exist stably in the environment [29]. The Δ*G* values of the three FQ molecular substitution pathways were all less than zero, which indicates that the substitution reactions occur spontaneously. Therefore, the inferred substitution reaction pathways are reasonable. That is, all three derivatives designed based on the 3D-QSAR model can be formed spontaneously. The order of difficulty of the substitution reactions between the substituents and the PAZ molecule is –CH_3_ > –H > –C_2_H_5_.

### 3.5. Risk Assessment of FQ Derivatives

#### 3.5.1. Human Health Risk Assessment of FQ Derivatives at Median Lethal Concentrations in Water

The exposure parameters given in this paper are partly from the median lethal concentrations (LC_50_) calculated by EPI software for fish, water fleas, and shrimp in the aquatic environment. They also partly refer to the Technical Guidelines for Risk Assessment of Polluted Sites (draft for comments) from the Ministry of Environmental Protection, China, and the recommended values in the Exposure Factor Manual from the US Environmental Protection Agency (EPA). The US EPA adopts a linear non-threshold model to describe the health effects of all carcinogens; that is, it assumes that any dose will produce some carcinogenic risks. This paper refers to the recommended values of the reference dose (RfD) of non-carcinogens and carcinogenic slope (SF) of carcinogens from the US EPA Integrated Risk Information Database for the evaluation of health risks. The specific evaluation results are shown in Table 8.

The non-carcinogenic risk assessments of the three FQs derivatives for human health were evaluated in water. The non-carcinogenic risks of the three derivatives under various conditions (sensitive, non-sensitive, carcinogenic, and non-carcinogenic) of various organisms (fish, water fleas, and shrimp) in water were less than one, indicating that the risk was acceptable.

Carcinogenic risk assessment of the three FQs derivatives to human health in water showed that the carcinogenic risk of derivative-1 was less than 1 × 10^−6^ in non-sensitive and non-carcinogenic media, which indicates that the risk can be neglected. Derivative-3 could also be neglected in sensitive, non-sensitive, and non-carcinogenic media. Except for the above, the carcinogenic risks of the other molecules were greater than 1 × 10^−6^ and less than 1 × 10^−6^, indicating that the risk is acceptable.

In conclusion, in both carcinogenic and non-carcinogenic risk assessments, the three derivatives pose negligible or acceptable risks to human health and aquatic organisms, and there is no possibility of unacceptable risk. Therefore, these three derivatives could be used for the production of new drugs.

#### 3.5.2. Risk Assessment of the Genotoxicities of Disinfection By-Products of the FQ Derivatives

**Figure 9:**
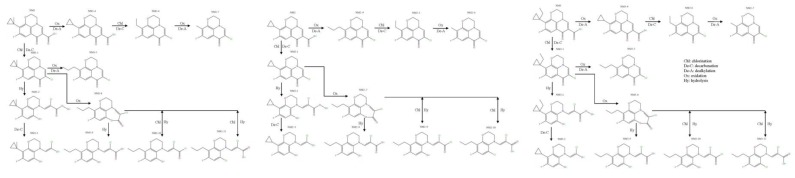
Conversion path of disinfection by-products of PAZ derivatives.

PAZ and its disinfection by-products in acidic, neutral, and alkaline environments were selected for analysis. The conversion reaction pathway (Figure 9) was considered to be the same as that of levofloxacin [30]. The known H-QSAR model of the genotoxicities of FQs was used to simulate and predict the disinfection by-products of PAZ. The predicted results from the FQ genotoxicity model were analyzed comprehensively. The average predicted values were selected as reference values.

The results for the FQs showed that the genotoxicity prediction values of all chlorine disinfection by-products of PAZ increased by 0.62% to 12.32% (Table 9). At the same time, the model predicted that the molecular genotoxicity of the chlorine disinfection by-products of derivative-2 decreased by 3.45% to 13.99%. The other two PAZ derivatives were also analyzed using the model (Table 10). Their disinfection by-products had some toxicity and posed a potential risk. In conclusion, the genetic toxicity risk of the designed PAZ derivatives after treatment in municipal wastewater treatment plants is significantly reduced compared with PAZ. At the same time, the genotoxicity prediction results of Cl-1, Cl-2, and Cl-3, which were chlorine disinfection by-products of derivative-1 and derivative-3, showed upward trends (Table 10). Although only two groups of data showed upward trends, whether the research and analysis can be carried out according to the above rules needs to be further expanded. Consequently, a follow-up study should be performed on disinfection by-products of antibiotics.

## 4. Conclusions

An improved model was established using a fuzzy comprehensive evaluation method with three common ADRs of FQs. After analyzing the results of the improved model, 48 FQ derivatives were designed, with lower ADRs than the original molecule. An evaluation of POP characteristics showed that the genotoxicities and photodegradation of the three FQs derivatives improved to varying degrees compared with the original molecule, and the bioconcentration was negligible. Three single-effect 3D-QSAR models of the FQs were constructed to validate the improved model and showed that it was reasonable to apply a weighting according to the incidences of the three common ADRs of FQs. The weighting used for each ADR was also appropriate. At the same time, the rationality of the multi-factor 3D-QSAR model of FQs based on the fuzzy evaluation method was verified. In the absence of complementary biological studies of these adverse drug reactions, the results reported here may be quite divergent from those found in humans or experimental animals in vivo. One major reason for this is that many adverse drug reactions are dependent upon enzyme-catalyzed metabolic activation (toxication) or on non-enzymatic conversion to toxic products and are not due to the parent drug moiety.

## Figures and Tables

**Table 1:** Docking and comprehensive evaluation of 29 fluoroquinolones (FQs)with three adverse drug reactions.

No.	Compounds	Docking Results			CEI
		3IP9	3N8V	2L0W	
1	Difloxacin	62.686	97.120	65.403	0.687
2	Enrofloxacin	64.070	90.401	90.476	0.707
3	Norfloxacin	58.422	90.557	88.709	0.601
4	Lomefloxacin	68.236	91.137	80.276	0.762
5	Ofloxacin	66.638	86.122	86.940	0.697
6	Pefloxacin	57.529	99.215	92.037	0.689
7	Fleroxacin	66.948	99.890	83.403	0.843
8	Ciprofloxacin	60.930	92.373	86.780	0.661
9	Bal ofloxacin	49.203	71.254	56.908	0.137
10	Marbofloxacin	65.300	95.242	88.670	0.777
11	Pipemidic acid	62.866	97.025	89.229	0.754
12	Cinoxacin	55.950	90.199	85.239	0.543
13	Enoxacin	60.660	86.605	87.601	0.596
14	Danofloxacin	67.775	91.772	93.715	0.797
15	Gatifloxacin	53.617	63.247	64.941	0.152
16	Levofloxacin	66.638	86.122	86.940	0.697
17	Rufloxacin	62.386	70.009	76.275	0.415
18	Pazufloxacin	74.075	94.849	86.206	0.925
19	Nadifloxacin	56.928	82.392	73.634	0.444
20	Moxifloxacin	46.442	73.108	73.101	0.152
21	Sparfloxacin	60.872	84.400	85.512	0.570
22	Sarafloxacin	59.595	82.206	72.443	0.487
23	Amifloxacin	59.512	86.682	80.072	0.555
24	Besifloxacin	55.541	79.981	76.735	0.401
25	Clinafloxacin	59.317	86.327	80.656	0.549
26	Grepafloxacin	66.245	90.285	93.474	0.753
27	Orbifloxacin	62.945	81.585	84.843	0.575
28	Sitafloxacin	60.702	72.267	83.135	0.428
29	Temafloxacin	66.934	88.733	75.241	0.699

**Table 2:** Evaluation parameters of the comparative molecular field analysis (CoMFA) models.

Model	*q* ^2^	*n*	*SEE*	*r* ^2^	*F*	*SEP*	*Q* ^2^ *_ext_*	*cSDEP*	*dq* ^2^ */dr* ^2^ *yy*	*r* ^2^ *_pred_*
CoMFA	0.560	10	0.007	0.999	1892.254	0.215	0.999	0.246	1.861	0.692

SEP: standard error of prediction; *cSDEP:* cross-validated standard error of prediction.

**Table 3:** Experimental and predicted values of fluoroquinolones through the CoMFA model.

No.	Compounds	CEI	Pred.	Residuals
1	Difloxacin^b^	0.687	0.688	0.18%
2	Enrofloxacin^a^	0.707	0.706	0.08%
3	Norfloxacin^a^	0.601	0.618	2.78%
4	Lomefloxacin^a^	0.762	0.756	0.82%
5	Ofloxacin^a^	0.697	0.702	0.76%
6	Pefloxacin^a^	0.689	0.686	0.39%
7	Fleroxacin^a^	0.843	0.846	0.35%
8	Ciprofloxacin^a^	0.661	0.663	0.26%
9	Balofloxacin^a^	0.137	0.140	1.92%
10	Marbofloxacin^a^	0.777	0.782	0.67%
11	Pipemidic acid^a^	0.754	0.751	0.36%
12	Cinoxacin^a^	0.543	0.542	0.23%
13	Enoxacin^a^	0.596	0.587	1.45%
14	Danofloxacin^a^	0.797	0.794	0.42%
15	Gatifloxacin^a^	0.152	0.149	1.74%
16	Levofloxacin^a^	0.697	0.700	0.47%
17	Rufloxacin^a^	0.415	0.417	0.50%
18	Pazufloxacin^a b^	0.925	0.922	0.28%
19	Nadifloxacin^a^	0.444	0.442	0.49%
20	Moxifloxacin^a^	0.152	0.152	0.23%
21	Sparfloxacin^a^	0.570	0.568	0.30%
22	Sarafloxacin^b^	0.487	0.471	3.31%
23	Amifloxacin^b^	0.555	0.554	0.22%
24	Besifloxacin^a^	0.401	0.327	18.49%
25	Clinafloxacin^b^	0.549	0.415	24.47%
26	Grepafloxacin^b^	0.753	0.544	27.74%
27	Orbifloxacin^a^	0.575	0.570	0.82%
28	Sitafloxacin^b^	0.428	0.462	8.01%
29	Temafloxacin^b^	0.699	0.676	3.26%

^a^ Training set; ^b^ Test set.

**Table 4:** Prediction of CEI index of derivatives based on CoMFA models.

Compounds	Structure	CEI	
		Pred.	Relative Error
No.18	PAZ	0.925	-
Derivative-1	1-Methyl-PAZ	0.619	−33.08%
Derivative-2	1-Hydrogen-PAZ	0.578	−37.51%
Derivative-3	1-Ethyl-PAZ	0.649	−29.84%
Derivative-4	1-Vinyl-PAZ	0.660	−28.65%
Derivative-5	1-Ethynyl-PAZ	0.719	−22.27%
Derivative-6	1-Carbonyl-PAZ	0.792	−14.38%
Derivative-7	5-Hydroxyl-PAZ	0.879	−4.97%
Derivative-8	5-Carboxyl-PAZ	0.827	−10.59%
Derivative-9	5-Sulfur-PAZ	0.848	−8.32%
Derivative-10	5-Fluorine-PAZ	0.814	−12.00%
Derivative-11	5-Chlorine-PAZ	0.717	−22.49%
Derivative-12	5-Bromine-PAZ	0.726	−21.51%
Derivative-13	1-Methyl-5-Hydroxyl-PAZ	0.762	−17.62%
Derivative-14	1-Methyl-5-Carboxyl-PAZ	0.743	−19.68%
Derivative-15	1-Methyl-5-Sulfur-PAZ	0.778	−15.89%
Derivative-16	1-Methyl-5-Fluorine-PAZ	0.715	−22.70%
Derivative-17	1-Methyl-Chlorine-PAZ	0.743	−19.68%
Derivative-18	1-Methyl-5-Bromine-PAZ	0.775	−16.22%
Derivative-19	1-Hydrogen-5-Hydroxyl-PAZ	0.652	−29.51%
Derivative-20	1-Hydrogen-5-Carboxyl-PAZ	0.592	−36.00%
Derivative-21	1-Hydrogen-5-Sulfur-PAZ	0.641	−30.70%
Derivative-22	1-Hydrogen-5-Fluorine-PAZ	0.549	−40.65%
Derivative-23	1-Hydrogen-Chlorine-PAZ	0.637	−31.14%
Derivative-24	1-Hydrogen-5-Bromine-PAZ	0.659	−28.76%
Derivative-25	1-Ethyl-5-Hydroxyl-PAZ	0.754	−18.49%
Derivative-26	1-Ethyl-5-Carboxyl-PAZ	0.796	−13.95%
Derivative-27	1-Ethyl-5-Sulfur-PAZ	0.776	−16.11%
Derivative-28	1-Ethyl-5-Fluorine-PAZ	0.727	−21.41%
Derivative-29	1-Ethyl-Chlorine-PAZ	0.828	−10.49%
Derivative-30	1-Ethyl-5-Bromine-PAZ	0.732	−20.86%
Derivative-31	1-Vinyl-5-Hydroxyl-PAZ	0.677	−26.81%
Derivative-32	1-Vinyl-5-Carboxyl-PAZ	0.613	−33.73%
Derivative-33	1-Vinyl-5-Sulfur-PAZ	0.710	−23.24%
Derivative-34	1-Vinyl-5-Fluorine-PAZ	0.691	−25.30%
Derivative-35	1-Vinyl-5-Chlorine-PAZ	0.558	−39.68%
Derivative-36	1-Vinyl-5-Bromine-PAZ	0.454	−50.92%
Derivative-37	1-Ethynyl-5-Hydroxyl-PAZ	0.659	−28.76%
Derivative-38	1-Ethynyl-5-Carboxyl-PAZ	0.880	−4.86%
Derivative-39	1-Ethynyl-5-Sulfur-PAZ	0.875	−5.41%
Derivative-40	1-Ethynyl-5-Fluorine-PAZ	0.742	−19.78%
Derivative-41	1- Ethynyl-5-Chlorine-PAZ	0.800	−13.51%
Derivative-42	1-Ethynyl-5-Bromine-PAZ	0.636	−31.24%
Derivative-43	1-Carbonyl-5-Hydroxyl-PAZ	0.718	−22.38%
Derivative-44	1-Carbonyl-5-Carboxyl-PAZ	0.682	−26.27%
Derivative-45	1-Carbonyl-5-Sulfur-PAZ	0.574	−37.95%
Derivative-46	1-Carbonyl-5-Fluorine-PAZ	0.600	−35.14%
Derivative-47	1-Carbonyl-5-Chlorine-PAZ	0.758	−18.05%
Derivative-48	1-Carbonyl-5-Bromine-PAZ	0.633	−31.57%

**Table 5:** Evaluation of POPs characteristics of PAZ derivatives based on constructed QSAR.

Compounds	pLOEC (Pred. AVG) *		log*t*_1/2_		log*K_ow_*	
	Pred.	Relative Error	Pred.	Relative Error	Pred.	Relative Error
PAZ	7.048	-	1.789	-	1.140	-
Derivative-1	7.404	5.05%	1.925	7.60%	0.191	−83.25%
Derivative-2	7.575	7.48%	1.954	9.22%	0.140	−87.72%
Derivative-3	7.322	3.89%	1.937	8.27%	0.289	−74.65%
Derivative-4	6.930	−1.67%	1.934	8.11%	1.689	48.16%
Derivative-5	6.936	−1.59%	1.869	4.47%	1.602	40.53%
Derivative-6	6.777	−3.85%	1.926	7.66%	1.254	10.00%
Derivative-7	7.082	0.48%	1.726	−3.52%	1.169	2.54%
Derivative-8	7.203	2.20%	1.821	1.79%	1.631	43.07%
Derivative-9	7.073	0.35%	1.728	−3.41%	1.354	18.77%
Derivative-10	7.089	0.58%	1.744	−2.52%	1.113	−2.37%
Derivative-11	7.031	−0.24%	1.732	−3.19%	1.170	2.63%
Derivative-12	7.033	−0.21%	1.954	9.22%	1.126	−1.23%
Derivative-13	7.022	−0.37%	1.827	2.12%	1.856	62.81%
Derivative-14	7.009	−0.55%	1.555	−13.08%	2.623	130.09%
Derivative-15	7.048	0.00%	1.987	11.07%	2.083	82.72%
Derivative-16	7.061	0.18%	1.831	2.35%	1.848	62.11%
Derivative-17	6.993	−0.78%	1.930	7.88%	1.907	67.28%
Derivative-18	7.007	−0.58%	1.862	4.08%	2.102	84.39%
Derivative-19	7.193	2.06%	1.860	3.97%	1.629	42.89%
Derivative-20	7.245	2.80%	1.942	8.55%	2.108	84.91%
Derivative-21	7.185	1.94%	1.862	4.08%	1.794	57.37%
Derivative-22	7.129	1.15%	1.952	9.11%	1.768	55.09%
Derivative-23	7.121	1.04%	1.969	10.06%	1.545	35.53%
Derivative-24	7.173	1.77%	1.877	4.92%	1.639	43.77%
Derivative-25	7.030	−0.26%	2.028	13.36%	2.055	80.26%
Derivative-26	7.034	−0.20%	2.073	15.87%	2.254	97.72%
Derivative-27	7.051	0.04%	1.978	10.56%	2.087	83.07%
Derivative-28	7.033	−0.21%	1.837	2.68%	1.875	64.47%
Derivative-29	6.988	−0.85%	1.956	9.33%	2.085	82.89%
Derivative-30	7.055	0.10%	1.847	3.24%	1.917	68.16%
Derivative-31	6.987	−0.87%	1.903	6.37%	2.317	103.25%
Derivative-32	6.938	−1.56%	1.414	−20.96%	2.378	108.60%
Derivative-33	6.743	−4.33%	1.769	−1.12%	2.599	127.98%
Derivative-34	7.253	2.91%	1.438	−19.62%	0.486	−57.37%
Derivative-35	7.242	2.75%	1.941	8.50%	2.403	110.79%
Derivative-36	7.293	3.48%	1.939	8.38%	2.330	104.39%
Derivative-37	7.123	1.06%	1.884	5.31%	2.205	93.42%
Derivative-38	6.949	−1.40%	1.709	−4.47%	2.515	120.61%
Derivative-39	6.922	−1.79%	2.060	15.15%	2.352	106.32%
Derivative-40	6.884	−2.33%	1.749	−2.24%	2.215	94.30%
Derivative-41	7.056	0.11%	1.854	3.63%	1.959	71.84%
Derivative-42	7.076	0.40%	1.942	8.55%	2.184	91.58%
Derivative-43	7.180	1.87%	1.880	5.09%	1.580	38.60%
Derivative-44	7.057	0.13%	1.870	4.53%	1.960	71.93%
Derivative-45	7.149	1.43%	1.864	4.19%	1.535	34.65%
Derivative-46	7.155	1.52%	1.953	9.17%	1.614	41.58%
Derivative-47	6.953	−1.35%	1.767	−1.23%	1.919	68.33%
Derivative-48	7.152	1.48%	1.921	7.38%	1.669	46.40%

* H-QSAR model chosen Pred. AVG as the final prediction value.

**Table 6:** Contrastive validation of single-factor and multi-factor adverse drug reactions of FQs derivatives.

Evaluation Project	PAZ	Derivative-1	Derivative-2	Derivative-3
Comprehensive evaluation index	0.925	0.619	0.578	0.649
		−33.08%	−37.51%	−29.84%
Convulsive Toxicity	74.075	62.218	60.048	63.499
		−16.01%	−18.94%	−14.28%
CEI × 50%	-	−16.54%	−18.76%	−14.92%
Relative error		3.23%	0.96%	4.30%
Gastrointestinal Toxicity	99.890	87.029	85.286	88.230
		−12.88%	−14.62%	−11.67%
CEI × 40%	-	−13.23%	−15.01%	−11.94%
Relative error		2.70%	2.57%	2.20%
Cardiotoxicity	93.715	91.282	91.352	91.266
		−2.60%	−2.52%	−2.61%
CEI × 10%	-	−3.31%	−3.75%	−2.98%
Relative error		15.07%	32.78%	12.42%

**Table 7:** PAZ derivatives’ three substitution reaction paths, positive frequency, Gibbs free energy, and energy barrier calculation.

Compounds	Frequency/(cm^−1^)	ΔG/(a.u.)	ΔE/(a.u.)
Path 1 (–CH_3_)	35.02	−0.026	39.307
Path 2 (–H)	39.97	−0.023	39.439
Path 3 (–C_2_H_5_)	35.60	−0.023	40.617

**Table 8:** Exposure dose *(ADD)*, Non-carcinogenic risk index *(HI)*, and Lifetime cancer risk index *(RI)* evaluation results of three PAZ derivatives.

Molecule	Medium	Carcinogenicity	Fish			Water Fleas			Shrimp		
			*ADD*	*HI*	*RI*	*ADD*	*HI*	*RI*	*ADD*	*HI*	*RI*
Derivative-1	sensitive	Carcinogenic	0.0002	0.0117	2.81 × 10^−5^	0.0001	0.0055	1.32 × 10^−5^	4.36 × 10^−5^	0.0022	5.23 × 10^−6^
		Non-Carcinogenic	8.03 × 10^−5^	0.0040	9.64 × 10^−6^	3.77 × 10^−5^	0.0019	4.53 × 10^−6^	1.49 × 10^−5^	0.0007	1.79 × 10^−6^
	Non-sensitive	Carcinogenic	9.41 × 10^−5^	0.0047	1.13 × 10^−5^	4.42 × 10^−5^	0.0022	5.30 × 10^−6^	1.75 × 10^−5^	0.0009	2.10 × 10^−6^
		Non-Carcinogenic	3.36 × 10^−5^	0.0017	4.03 × 10^−6^	1.58 × 10^−5^	0.0008	1.89 × 10^−6^	6.25 × 10^−6^	0.0003	7.50 × 10^−7^
Derivative-2	sensitive	Carcinogenic	0.0005	0.0248	5.94 × 10^−5^	0.0003	0.0134	3.21 × 10^−5^	0.0001	0.0067	1.60 × 10^−5^
		Non-Carcinogenic	0.0002	0.0085	2.04 × 10^−5^	9.19 × 10^−5^	0.0046	1.10 × 10^−5^	4.58 × 10^−5^	0.0023	5.50 × 10^−6^
	Non-sensitive	Carcinogenic	0.0002	0.0100	2.39 × 10^−5^	0.0001	0.0054	1.29 × 10^−5^	5.37 × 10^−5^	0.0027	6.44 × 10^−6^
		Non-Carcinogenic	7.11 × 10^−5^	0.0036	8.53 × 10^−6^	3.85 × 10^−5^	0.0019	4.61 × 10^−6^	1.92 × 10^−5^	0.0010	2.30 × 10^−6^
Derivative-3	sensitive	Carcinogenic	0.0001	0.0052	1.21 × 10^−5^	4.17 × 10^−5^	0.0021	5.00 × 10^−6^	1.29 × 10^−5^	0.0006	1.55 × 10^−6^
		Non-Carcinogenic	3.55 × 10^−5^	0.0018	4.26 × 10^−6^	1.43 × 10^−5^	0.0007	1.72 × 10^−6^	4.41 × 10^−6^	0.0002	5.30 × 10^−7^
	Non-sensitive	Carcinogenic	4.16 × 10^−5^	0.0021	4.99 × 10^−6^	1.68 × 10^−5^	0.0008	2.01 × 10^−6^	5.17 × 10^−6^	0.0003	6.21 × 10^−7^
		Non-Carcinogenic	1.48 × 10^−5^	0.0007	1.78 × 10^−6^	5.98 × 10^−6^	0.0003	7.18 × 10^−7^	1.85 × 10^−6^	9.24 × 10^−5^	2.22 × 10^−7^

**Table 9:** Risk assessment of genotoxicity of disinfection by-products of PAZ.

Compounds	Pred.59	Relative Error	Pred.61	Relative Error	Pred. AVG *	Relative Error
PAZ	7.048					
PAZ-Cl-1	7.448	5.68%	7.992	13.39%	7.720	9.53%
PAZ-Cl-2	7.719	9.52%	8.113	15.11%	7.916	12.32%
PAZ-Cl-3	7.806	10.75%	7.991	13.38%	7.899	12.07%
PAZ-Cl-4	7.588	7.66%	8.195	16.27%	7.891	11.96%
PAZ-Cl-5	7.378	4.68%	7.755	10.03%	7.567	7.36%
PAZ-Cl-6	7.275	3.22%	7.678	8.94%	7.477	6.09%
PAZ-Cl-7	7.149	1.43%	7.533	6.88%	7.341	4.16%
PAZ-Cl-8	6.967	−1.15%	7.235	2.65%	7.101	0.75%
PAZ-Cl-9	6.829	−3.11%	7.355	4.36%	7.092	0.62%
PAZ-Cl-10	7.497	6.37%	7.709	9.38%	7.603	7.87%
PAZ-Cl-11	7.481	6.14%	7.670	8.83%	7.575	7.48%
PAZ-Cl-12	7.415	5.21%	7.649	8.53%	7.532	6.87%
PAZ-Cl-13	7.375	4.64%	7.572	7.43%	7.473	6.03%

* H-QSAR model chosen Pred. AVG as the final prediction value.

**Table 10:** Risk assessment of genotoxicity of disinfection by-products of PAZ derivatives.

Compounds	Pred.59	Relative Error	Pred.61	Relative Error	Pred. AVG *	Relative Error
Derivative-1	7.404					
Derivative-1-Cl-1	7.335	−0.93%	7.907	6.79%	7.621	2.93%
Derivative-1-Cl-2	7.439	0.47%	7.745	4.61%	7.592	2.54%
Derivative-1-Cl-3	7.523	1.61%	7.615	2.85%	7.569	2.23%
Derivative-1-Cl-4	7.162	−3.27%	7.466	0.84%	7.314	−1.22%
Derivative-1-Cl-5	6.506	−12.13%	6.524	−11.89%	6.515	−12.01%
Derivative-1-Cl-6	6.815	−7.96%	7.227	−2.39%	7.021	−5.17%
Derivative-1-Cl-7	6.823	−7.85%	7.200	−2.76%	7.011	−5.31%
Derivative-1-Cl-8	7.125	−3.77%	7.181	−3.01%	7.153	−3.39%
Derivative-1-Cl-9	7.076	−4.43%	7.119	−3.85%	7.097	−4.15%
Derivative-1-Cl-10	7.012	−5.29%	7.100	−4.11%	7.056	−4.70%
Derivative-1-Cl-11	7.027	−5.09%	7.024	−5.13%	7.025	−5.12%
Derivative-2	7.575					
Derivative-2-Cl-1	7.049	−6.94%	7.399	−2.32%	7.224	−4.63%
Derivative-2-Cl-2	7.145	−5.68%	7.293	−3.72%	7.219	−4.70%
Derivative-2-Cl-3	7.246	−4.34%	7.167	−5.39%	7.207	−4.86%
Derivative-2-Cl-4	6.506	−14.11%	6.524	−13.87%	6.515	−13.99%
Derivative-2-Cl-5	6.815	−10.03%	7.227	−4.59%	7.021	−7.31%
Derivative-2-Cl-6	6.823	−9.93%	7.200	−4.95%	7.011	−7.45%
Derivative-2-Cl-7	7.125	−5.94%	7.181	−5.20%	7.153	−5.57%
Derivative-2-Cl-8	7.076	−6.59%	7.119	−6.02%	7.097	−6.31%
Derivative-2-Cl-9	7.012	−7.43%	7.100	−6.27%	7.056	−6.85%
Derivative-2-Cl-10	7.027	−7.23%	7.024	−7.27%	7.025	−7.26%
Derivative-3	7.322					
Derivative-3-Cl-1	7.396	1.01%	7.951	8.59%	7.674	4.81%
Derivative-3-Cl-2	7.493	2.34%	7.811	6.68%	7.652	4.51%
Derivative-3-Cl-3	7.578	3.50%	7.683	4.93%	7.630	4.21%
Derivative-3-Cl-4	7.162	−2.19%	7.466	1.97%	7.314	−0.11%
Derivative-3-Cl-5	6.506	−11.14%	6.524	−10.90%	6.515	−11.02%
Derivative-3-Cl-6	6.815	−6.92%	7.227	−1.30%	7.021	−4.11%
Derivative-3-Cl-7	6.823	−6.82%	7.200	−1.67%	7.011	−4.25%
Derivative-3-Cl-8	7.125	−2.69%	7.181	−1.93%	7.153	−2.31%
Derivative-3-Cl-9	7.076	−3.36%	7.119	−2.77%	7.097	−3.07%
Derivative-3-Cl-10	7.012	−4.23%	7.100	−3.03%	7.056	−3.63%
Derivative-3-Cl-11	7.027	−4.03%	7.024	−4.07%	7.025	−4.06%

* H-QSAR model chosen Pred. AVG as the final prediction value.
